# A comparative analysis of the intrauterine transcriptome in fertile and subfertile mares using cytobrush sampling

**DOI:** 10.1186/s12864-021-07701-3

**Published:** 2021-05-22

**Authors:** Katharina S. Weber, Karen Wagener, Miguel Blanco, Stefan Bauersachs, Heinrich Bollwein

**Affiliations:** 1 Clinic of Reproductive Medicine, Department for Farm Animals, Vetsuisse Faculty Zurich, University of Zurich, Lindau (ZH), Switzerland; 2 Present address: Department for Farm Animals and Veterinary Public Health, Clinical Unit for Herd Health Management in Ruminants, University of Veterinary Medicine, Vienna, Austria; 3 The Lewitz Stud, Neustadt-Glewe, Germany; 4 Institute of Veterinary Anatomy, Vetsuisse Faculty Zurich, University of Zurich, Lindau (ZH), Switzerland

**Keywords:** Mare, Subfertility, Uterine transcriptome, Cytobrush, RNA-seq, Biomarker

## Abstract

**Background:**

Subfertility is a major problem in modern horse breeding. Especially, mares without clinical signs of reproductive diseases, without known uterine pathogens and no evidence of inflammation but not becoming pregnant after several breeding attempts are challenging for veterinarians. To obtain new insights into the cause of these fertility problems and aiming at improving diagnosis of subfertile mares, a comparative analysis of the intrauterine transcriptome in subfertile and fertile mares was performed. Uterine cytobrush samples were collected during estrus from 57 mares without clinical signs of uterine diseases. RNA was extracted from the cytobrush samples and samples from 11 selected subfertile and 11 fertile mares were used for Illumina RNA-sequencing.

**Results:**

The cytobrush sampling was a suitable technique to isolate enough RNA of high quality for transcriptome analysis. Comparing subfertile and fertile mares, 114 differentially expressed genes (FDR = 10%) were identified. Metascape enrichment analysis revealed that genes with lower mRNA levels in subfertile mares were related to ‘extracellular matrix (ECM)’, ‘ECM-receptor interaction’, ‘focal adhesion’, ‘immune response’ and ‘cytosolic calcium ion concentration’, while DEGs with higher levels in subfertile mares were enriched for ‘monocarboxyl acid transmembrane transport activity’ and ‘protein targeting’.

**Conclusion:**

Our study revealed significant differences in the uterine transcriptome between fertile and subfertile mares and provides leads for potential uterine molecular biomarkers of subfertility in the mare.

**Supplementary Information:**

The online version contains supplementary material available at 10.1186/s12864-021-07701-3.

## Background

Subfertility represents a substantial problem for the horse breeding industry [1] as it leads to high economic losses for the owners. Subfertile mares do either not conceive or require more examinations, inseminations and treatments to get pregnant than their fertile counterparts. Many factors such as age, reproductive status, gynecological health of the mare, sperm quality, sperm preservation and breeding management have an effect on fertility [2–4]. Clinical endometritis is one of the most common causes for fertility problems in mares [1] and was ranked in the top three medical problems in equine adult patients [5]. Endometritis can be divided into acute infectious, chronic infectious or non-infectious endometritis. The most common types of endometritis in mares are bacterially infectious endometritis and persistent breeding induced endometritis (PBIE) [6, 7]. Mares susceptible to PBIE show prolonged persistent post breeding uterine inflammation, interfering with the arrival of the embryo in the uterus 5–6 days after breeding [8]. Mares with endometritis have a lower conception rate and a higher risk for early embryonic death and mid-gestational abortion. Clinical signs of endometritis include intrauterine fluid, excessive pattern of endometrial edema, vaginitis, vaginal discharge, abnormal estrous cycles and cervicitis. Often endometritis can be diagnosed by detecting clinical signs, uterine inflammation in cytological examination or pathogens in uterine microbial culture [9]. However, there are also mares which don’t get pregnant after several breeding attempts with sperm of fertile stallions without showing any pathological signs using these diagnostic methods. Le Blanc and Causey [9] described these disturbances in fertility as hidden cases of endometritis or subclinical endometritis.

Although in many studies the histological examination of uterine biopsy samples was considered as the gold standard for diagnosis endometritis [10–13] and for predicting fertility by using the Kenney and Doig score [14], in practice, currently mostly double-guarded uterine swabs for microbial culture and cytobrushes for cytology are used, as these methods are less invasive than the biopsy and less time consuming than histological examination. The sensitivity of microbial culture and cytology is low and these diagnostic methods have a high incidence of false negative results [6, 10, 13, 15]. Many bacteria are difficult to cultivate in vitro and are therefore not detectable by classical bacteriology [16–18]. Moreover, some bacteria, e.g. gram negative bacteria like *Escherichia coli* don’t induce a cellular immunological reaction with a high amount of neutrophils detected by the cytological examination in contrast to other bacteria, such as *Streptococci* [1, 19]. Therefore, for mares without clinical signs of uterine diseases, without known pathogens in culture, no evidence of inflammation in cytology but not becoming pregnant after several breeding attempts more accurate diagnostic methods are needed to predict fertility.

It seems likely that underlying mechanisms for subfertility can be found at the molecular level. For instance mares susceptible to persistent endometritis show differences in innate immune response to insemination [8, 20–22] and induced infectious endometritis [23] compared to resistant mares at mRNA expression level. The mRNA expression of pro- inflammatory cytokines (*IL6, IL1RN, IL1B, CXCL8*), anti-inflammatory cytokines (*IL10*), tumor necrosis factor (*TNF*), C-C motif chemokine ligand 2 (*CCL2*), antimicrobial peptides, secreted phospholipase A2 (*PLA2G2A*), lipocalin 2 (*LCN2*) and lactotransferrin (*LTF*) differ between susceptible and resistant mares [8, 20–23]. Recently, it has been shown that mares susceptible for PBIE show a different expression pattern of genes associated with innate immunity even before breeding and that antimicrobial peptides equine b-defensin 1 (*DEFB1*), lysozyme (*LYZ*) and secretory leukoprotease inhibitor (*SLPI*) can be used as diagnostic marker for susceptibility [22].

Gene expression profiling of the healthy, receptive equine endometrium has shown that the transcriptome differed among estrous cycle stages [24, 25]. Genes upregulated during estrus were associated with extracellular matrix related categories and immune regulated functions [24, 25]. These physiological changes in uterine gene expression could play an important role in successful reproduction. For instance, the uterine immune system may prepare the uterus for potential foreign material ascending through the open cervix during estrus by upregulation of genes related to immune response [24, 25].

In recent years, gene expression analysis has been applied in several studies to identify genes and their networks associated with receptivity of the human endometrium at the time of implantation by comparing women with recurrent miscarriage [26, 27] or recurrent implantation failure [26, 28–30] and fertile women. Furthermore, in cows, different studies were performed to identify endometrial gene expressions related to fertility [31–35]. However, to our knowledge, no study investigated yet the relationship between the equine uterine transcriptome and fertility in mares using cytobrush samples collected during estrus.

In most of the equine and human studies uterine biopsy samples were taken for transcriptome and mRNA analysis, while in cattle cytobrush samples were often used for mRNA analysis. In different bovine studies, it was shown that cytobrush sampling provides a much less invasive method to isolate RNA of sufficient quantity and quality for gene expression analysis [31, 36] compared to the biopsy of the endometrium.

With the aim to improve the diagnosis of subfertile mares without clinical signs of uterine diseases and to characterize RNA markers to predict fertility, our objective was to perform a comparative analysis of the intrauterine transcriptome at estrus of fertile and subfertile mares without clinical signs of uterine diseases. A second objective was to investigate the suitability of samples collected by cytobrush from the equine uterus for transcriptome analysis.

## Results

### Cytology and bacteriology

The cytological examination did not reveal an intrauterine inflammation at the time of sampling in all mares. Bacteria were detected in 33 of 57 mares (57.9%) in microbial culture. Facultative pathogens were obtained in 12 of 57 mares (21.1%). These 12 samples with facultative pathogens were excluded from further analysis. From each group of the fertile mares (FB-P) and subfertile mares (RB-N) 11 mares without facultative pathogens were selected for RNA sequencing.

### Isolation of RNA from cytobrush samples and Illumina RNA-sequencing

The cytobrush sampling was a suitable technique to isolate enough RNA of high quality for transcriptome analysis. The concentration of the total RNA was between 40 and 669 ng/μl, while the A260/A280 ratio was between 1.95 and 2.09. The obtained RNA integrity numbers (RIN) ranged from 8.9 to 10 in all 57 samples.

The RNA sequencing results revealed after filtering of the fastq files library sizes between 10.9 and 30.9 million reads per sample with an average of 18.4 million reads. After filtering genes with low read counts, in total 15,318 different genes were detectable and used for differential gene expression analysis.

### Identification of differentially expressed genes

**Fig. 1:**
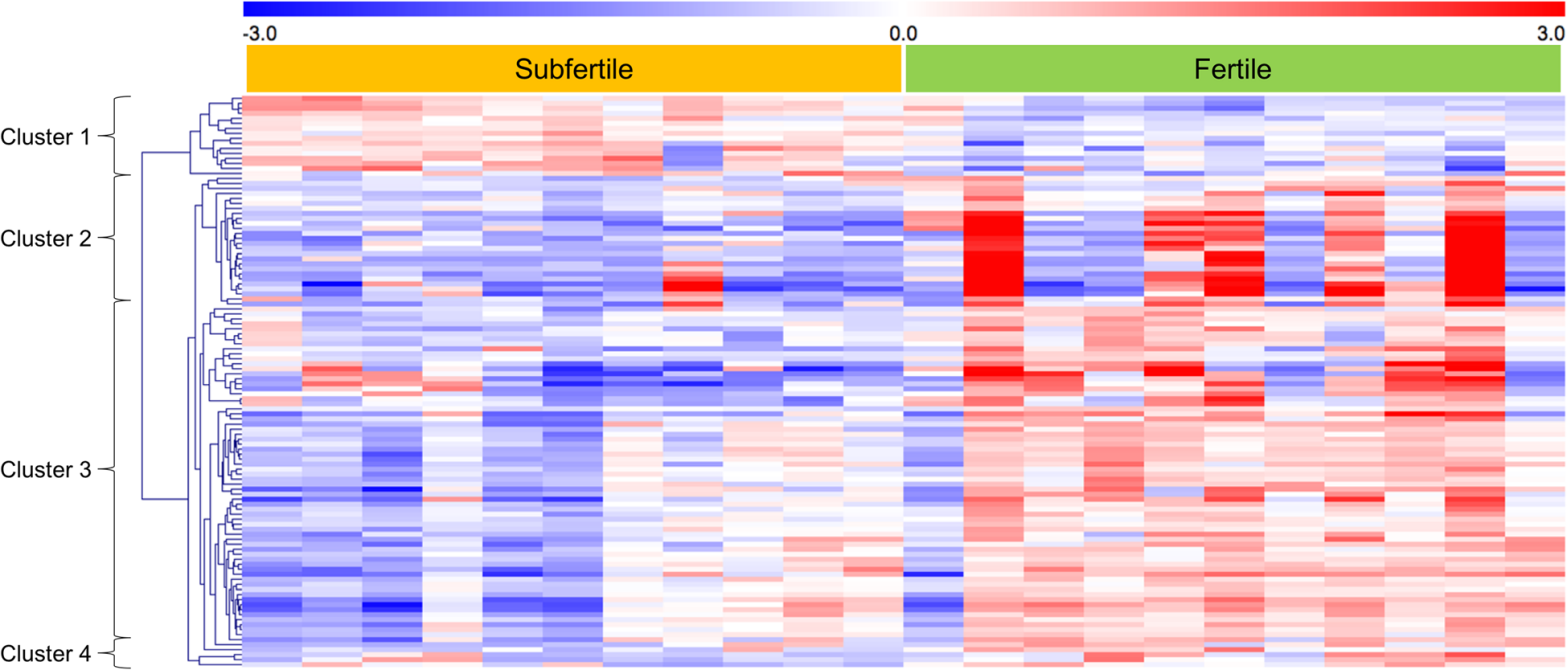
Heat map and hierarchical cluster analysis of DEGs between subfertile and fertile mares (FDR < 0.1). Each row represents 1 DEG, each column 1 sample. Red color represents higher and blue color lower expression of the gene compared to the mean of all samples (mean-centered values in log2 scale from −3 to 3)

The intrauterine transcriptome differed between subfertile and fertile mares without clinical signs of uterine diseases. Using Edge R analysis, 114 genes were found as differentially expressed between subfertile and fertile mares (FDR < 0.1; Fig. 1) (Additional file 1). Ninety-eight genes were significantly downregulated and 16 genes upregulated in subfertile mares compared to fertile mares. The expression of neuromedin U (*NMU*), synaptogamin 12 (*SYT12*), uncharacterized *LOC111767890*, UL16 binding protein 1 (*LOC100063831*) were decreased to the greatest extent, while the expression of solute carrier family 10 member 2 (*SLC10A2*), 40S ribosomal protein S2-like (*LOC100147232*) and 60S ribosomal protein L26-like (*LOC10052427*) were increased to the greatest extent in subfertile mares compared to fertile mares.

Hierarchical cluster analysis of the DEGs revealed a separation of DEGs upregulated (cluster 1) or downregulated (clusters 2, 3, 4) in samples derived from subfertile mares (Fig. 1). The downregulated genes were separated in three clusters. Cluster 2 showed DEGs with increased expression in only 5 of the fertile mares. Differences in cluster 3 and 4 were more consistent above all samples. A few samples of the subfertile and fertile group, respectively, showed expression patterns in part more similar to the respective other group. The DEGs of cluster 1 and the DEGs of clusters 3 and 4 are listed in Tables 1 and 2, respectively.

**Table 1:** DEGs of Cluster1: DEGs upregulated in subfertile mares compared to fertile mares

Gene symbol	Entrez Gene ID	Gene description	Human gene symbol	log2 FC SUB/FER	*P*-value	FDR
*SLC10A2*	100051264	solute carrier family 10 member 2	*SLC10A2*	1.82	0.0002	0.0591
*LOC100147232*	100147232	40S ribosomal protein S2 like		1.80	0.0000	0.0012
*LOC100052427*	100052427	60S ribosomal protein L26-like		1.64	0.0000	0.0200
*LOC100051778*	100051778	60S ribosomal protein L21	*RPL21*	1.63	0.0000	0.0035
*LOC100065786*	100065786	40S ribosomal protein S17	*RPS17*	1.46	0.0000	0.0014
*LOC100067178*	100067178	Mesothelin	*MSLN*	1.13	0.0005	0.0861
*SLC16A9*	100062703	solute carrier family 16 member 9	*SLC16A9*	0.98	0.0001	0.0417
*ELOVL2*	100063624	ELOVL fatty acid elongase 2	*ELOVL2*	0.96	0.0001	0.0474
*LOC111767704*	111767704	uncharacterized LOC111767704		0.91	0.0000	0.0179
*CRYL1*	100054141	crystallin lambda 1	*CRYL1*	0.89	0.0001	0.0338
*CTSE*	100055161	cathepsin E	*CTSE*	0.82	0.0002	0.0591
*LOC100072143*	100,072,143	centrin-4		0.80	0.0001	0.0318
*SLC16A5*	100060017	solute carrier family 16 member 5	*SLC16A5*	0.57	0.0005	0.0861
*TOMM7*	100630688	translocase of outer mitochondrial membrane 7	*TOMM7*	0.52	0.0002	0.0591
*MYCBP*	100068904	MYC binding protein	*MYCBP*	0.47	0.0002	0.0564
*SDHAF4*	100629833	succinate dehydrogenase complex assembly factor 4	*SDHAF4*	0.45	0.0004	0.0763

**Table 2:** DEGs of Clusters 3 and 4: DEGs downregulated in subfertile mares compared to fertile mares

Gene symbol	Entrez Gene ID	Gene description	Human gene symbol	log2 FC SUB/FER	*P*-value	FDR
*ACAP1*	100072970	ArfGAP with coiled-coil, ankyrin repeat and PH domains 1	*ACAP1*	−1.01	0.0000	0.0291
*ACKR3*	100057501	atypical chemokine receptor 3	*ACKR3*	−1.60	0.0001	0.0474
*ADAMTS7*	100059959	ADAM metallopeptidase with thrombospondin type 1 motif 7	*ADAMTS7*	−1.39	0.0001	0.0318
*AKR1E2*	100070632	aldo-keto reductase family 1 member E2	*AKR1E2*	−1.93	0.0000	0.0179
*ANKRD10*	100066458	ankyrin repeat domain 10	*ANKRD10*	−0.66	0.0007	0.0998
*ANO8*	100146761	anoctamin 8	*ANO8*	−1.24	0.0004	0.0763
*APBA3*	100146445	amyloid beta precursor protein binding family A member 3	*APBA3*	−0.62	0.0003	0.0708
*C1QA*	100058097	complement C1q A chain	*C1QA*	−0.66	0.0006	0.0861
*C1QB*	100071667	complement C1q B chain	*C1QB*	−0.74	0.0002	0.0562
*CEP131*	100056159	centrosomal protein 131	*CEP131*	−0.85	0.0002	0.0529
*CIAO3*	100065271	cytosolic iron-sulfur assembly component 3	*CIAO3*	−0.57	0.0007	0.0998
*CLK1*	100067832	CDC like kinase 1	*CLK1*	−0.94	0.0004	0.0796
*CLK2*	100063546	CDC like kinase 2	*CLK2*	−0.69	0.0006	0.0861
*COL16A1*	100056083	collagen type XVI alpha 1 chain	*COL16A1*	−1.72	0.0005	0.0847
*COL4A1*	100066148	collagen type IV alpha 1 chain	*COL4A1*	−1.16	0.0003	0.0658
*COL4A2*	100066264	collagen type IV alpha 2 chain	*COL4A2*	−1.17	0.0001	0.0354
*COL6A1*	100050035	collagen type VI alpha 1 chain	*COL6A1*	−1.84	0.0006	0.0897
*CYTH4*	100069735	cytohesin 4	*CYTH4*	−0.79	0.0004	0.0798
*DENND1C*	100065730	DENN domain containing 1C	*DENND1C*	−0.91	0.0006	0.0861
*DLG4*	100061544	discs large MAGUK scaffold protein 4	*DLG4*	−0.98	0.0003	0.0643
*DNASE1L3*	100057863	deoxyribonuclease 1 like 3	*DNASE1L3*	−1.66	0.0001	0.0474
*EHBP1L1*	100057282	EH domain binding protein 1 like 1	*EHBP1L1*	−0.93	0.0001	0.0417
*ENTPD6*	100057043	ectonucleoside triphosphate diphosphohydrolase 6	*ENTPD6*	−0.70	0.0003	0.0620
*FER1L5*	100062182	fer-1 like family member 5	*FER1L5*	−1.77	0.0002	0.0591
*FN1*	100034189	fibronectin 1	*FN1*	−2.21	0.0001	0.0472
*GRAMD1B*	100063638	GRAM domain containing 1B	*GRAMD1B*	−0.61	0.0004	0.0763
*IRF8*	100056218	interferon regulatory factor 8	*IRF8*	−0.79	0.0005	0.0861
*JAK3*	100147451	Janus kinase 3	*JAK3*	−0.66	0.0003	0.0673
*KIF7*	100069672	kinesin family member 7	*KIF7*	−0.82	0.0006	0.0867
*LAT*	100064430	linker for activation of T cells	*LAT*	−0.93	0.0001	0.0417
*LLGL1*	100051856	LLGL1, scribble cell polarity complex component	*LLGL1*	−0.50	0.0006	0.0861
*LOC100054029*	100054029	leukocyte immunoglobulin-like receptor subfamily A member 5		−1.26	0.0006	0.0883
*LOC100054448*	100054448	saoe class I histocompatibility antigen, A alpha chain	*HLA-A*	−1.26	0.0000	0.0024
*LOC100055483*	100055483	Ig mu chain C region membrane-bound form-like	*IGHM*	−1.82	0.0003	0.0609
*LOC100063097*	100063097	mitotic-spindle organizing protein 2B-like	*MZT2B*	−1.70	0.0001	0.0423
*LOC100073089*	100073089	ectonucleotide pyrophosphatase/phosphodiesterase family member 3	*ENPP3*	−1.26	0.0000	0.0200
*LOC100629324*	100629324	uncharacterized LOC100629324	*MEG3*	−2.85	0.0001	0.0417
*LOC102149846*	102149846	immunoglobulin heavy constant gamma 1-like	*IGHG1*	−2.14	0.0005	0.0861
*LOC102150085*	102150085	immunoglobulin heavy constant gamma 1-like	*IGHG1*	−2.67	0.0000	0.0240
*LOC102150790*	102150790	uncharacterized LOC102150790		−1.44	0.0000	0.0179
*LOC106781059*	106781059	uncharacterized LOC106781059		−1.46	0.0001	0.0327
*LOC106781303*	106781303	immunoglobulin heavy constant alpha 2-like	*IGHA1*	−1.98	0.0005	0.0861
*LOC106781940*	106781940	uncharacterized LOC106781940		−1.45	0.0004	0.0762
*LOC106783330*	106783330	uncharacterized LOC106783330		−1.67	0.0001	0.0417
*LOC111767520*	111767520	uncharacterized LOC111767520		−1.60	0.0003	0.0669
*LOC111768661*	111768661	translation initiation factor IF-2-like		−1.19	0.0006	0.0897
*LOC111768809*	111768809	uncharacterized LOC111768809		−2.47	0.0000	0.0113
*LOC111771758*	111771758	GTPase IMAP family member 5-like		−0.92	0.0001	0.0474
*LOC111774331*	111774331	uncharacterized LOC111774331		−1.17	0.0003	0.0620
*MCF2L*	100067048	MCF.2 cell line derived transforming sequence like	*MCF2L*	−0.74	0.0001	0.0405
*MICAL1*	100066627	microtubule associated monooxygenase, calponin and LIM domain containing 1	*MICAL1*	−0.90	0.0000	0.0263
*MMP25*	100068942	matrix metallopeptidase 25	*MMP25*	−1.10	0.0003	0.0619
*NAAA*	100057831	N-acylethanolamine acid amidase	*NAAA*	−1.04	0.0003	0.0642
*NUP210L*	100056659	nucleoporin 210 like	*NUP210L*	−1.11	0.0002	0.0553
*PDE10A*	100050311	phosphodiesterase 10A	*PDE10A*	−2.44	0.0000	0.0195
*PLCB2*	100057315	phospholipase C beta 2	*PLCB2*	−0.93	0.0005	0.0861
*PLXNA3*	100058349	plexin A3	*PLXNA3*	−1.31	0.0005	0.0861
*PREX1*	100071328	phosphatidylinositol-3,4,5-trisphosphate dependent Rac exchange factor 1	*PREX1*	−0.68	0.0006	0.0861
*RAB44*	100629655	RAB44, member RAS oncogene family	*RAB44*	−1.29	0.0005	0.0861
*RYR1*	100034090	ryanodine receptor 1	*RYR1*	−1.31	0.0004	0.0737
*SLC8B1*	100056481	solute carrier family 8 member B1	*SLC8B1*	−0.66	0.0003	0.0676
*SNRNP70*	100054907	small nuclear ribonucleoprotein U1 subunit 70	*SNRNP70*	−0.74	0.0002	0.0591
*THBS2*	100050044	thrombospondin 2	*THBS2*	−3.13	0.0000	0.0294
*TIA1*	100050503	TIA1 cytotoxic granule associated RNA binding protein	*TIA1*	−0.55	0.0006	0.0861
*TNNT2*	100146343	troponin T2, cardiac type	*TNNT2*	−2.20	0.0001	0.0417
*TNXB*	100059315	tenascin XB	*TNXB*	−1.19	0.0000	0.0240
*TOP3B*	100051153	DNA topoisomerase III beta	*TOP3B*	−0.92	0.0002	0.0602
*TPCN2*	102150167	two pore segment channel 2	*TPCN2*	−0.76	0.0002	0.0591
*VGLL3*	100069930	vestigial like family member 3	*VGLL3*	−1.41	0.0006	0.0883
*WDR90*	100066920	WD repeat domain 90	*WDR90*	−0.75	0.0005	0.0861
*ZBP1*	100055754	Z-DNA binding protein 1	*ZBP1*	−0.96	0.0000	0.0294
*ZNF333*	100064631	zinc finger protein 333	*ZNF333*	−0.67	0.0007	0.0998

### Overrepresented functional categories

**Fig. 2:**
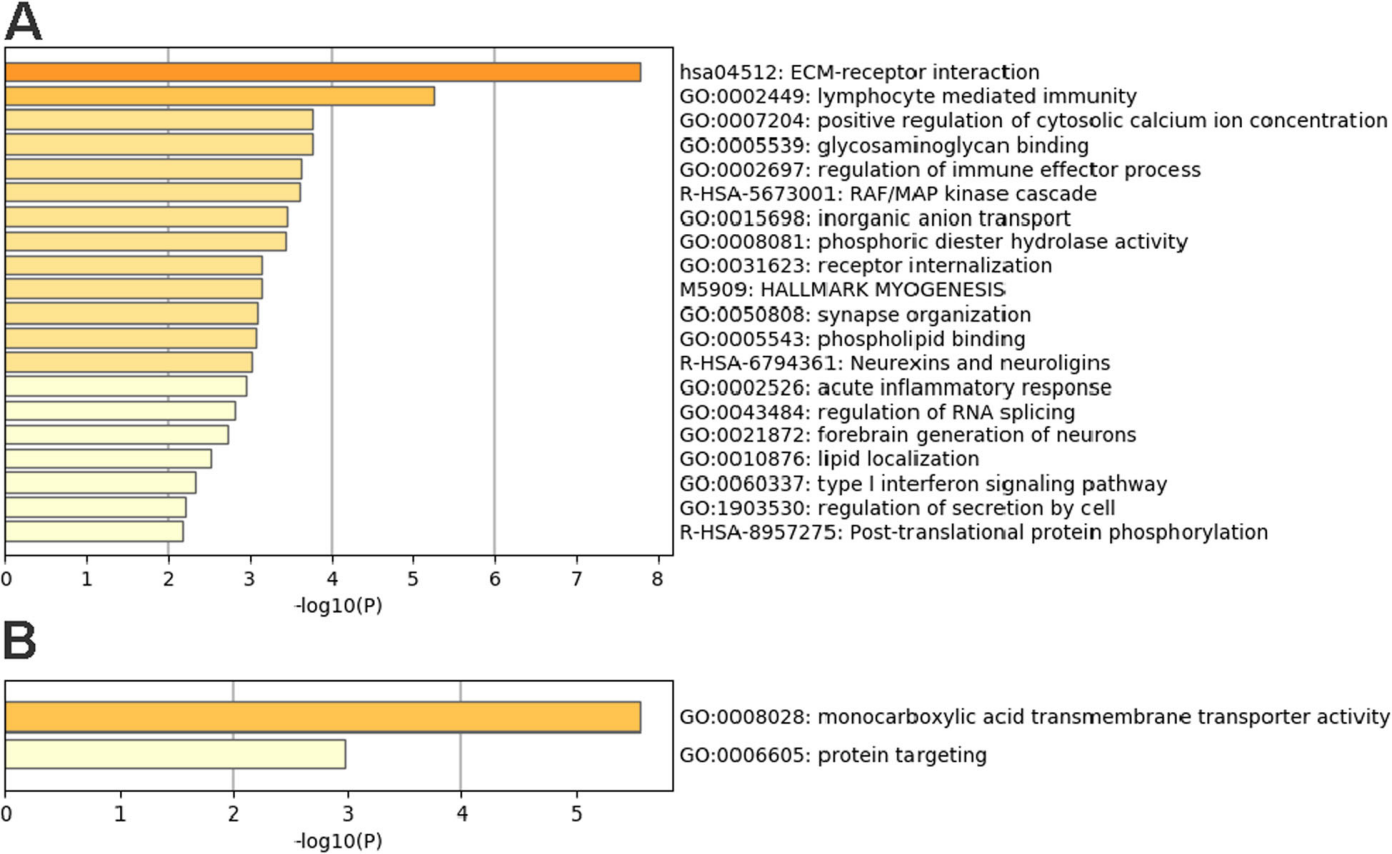
Metascape analysis: **a** Top 20 most enriched terms in genes downregulated in subfertile mares compared to fertile mares. **b** Enriched terms in genes upregulated in subfertile mares compared to fertile mares

The DEGs were analyzed for overrepresented functional categories and pathways in fertile or subfertile mares using the Metascape enrichment analysis tool (Table 3, Fig. 2, Additional file 2). The analyses were performed separately for genes upregulated or downregulated in subfertile mares compared to fertile mares, uploading the corresponding human NCBI Entrez gene IDs. Eighty-five genes of the downregulated genes and 12 of the upregulated genes could be assigned to a corresponding human gene symbol.

**Table 3:** Metascape functional term enrichment analysis of DEGs subfertile vs fertile mares

Most informative categories of Metascape enrichment analysis	Log_10_ (*P*-value)	Assigned genes
***Genes with lower expression in subfertile*** **vs.** ***fertile mares***		
Extracellular matrix, ECM-receptor interaction, Focal adhesion, Collagen trimer, PI3-Akt signaling pathway	−7.8	*COL4A1,COL4A2,COL6A1,FN1,ITGB3,THBS2,TNXB,COL16A1,C1QA,C1QB,ACHE,MMP25,FGFR1,JAK3,SLC39A8,ADAMTS7,PNPLA2,ANO8,DLG4,TIA1,PLCB2,PLXNA3,LLGL1,ACKR3,PDE2A,RYR1,ERFE,AKR1C4,CLK2*
Lymphocyte mediated immunity, complement activation, adaptive immune response	−4.4	*C1QA,C1QB,HLAA,IGHA1,IGHG1,IGHM,CLCF1,ULBP3,JAK3,FN1,DLG4,ENPP3,STAC,ACKR3,LAT,PREX1,IRF8,ITGB3*
Positive regulation of cytosolic calcium ion concentration, muscle contraction	−3.8	*DLG4,PLCB2,RYR1,NMU,ACKR3,SLC8B1,TPCN2,SLC39A8,ERFE,COL6A1,STAC,ITGB3,PNPLA2,FGFR1,SYT12,ATP8B2,ANO8,TNNT2*
Glycosaminoglycan binding	−3.8	*COL16A1,FGFR1,FN1,IGHM,THBS2,TNXB,ITGB3,PREX1,IGHA1,SLC8B1*
Regulation of immune effector process	−3.7	*C1QA,C1QB,DNASE1L3,HLA-A,IGHG1,JAK3,ENPP3, CLCF1,ULBP3*
Peptidyl-tyrosine phosphorylation	−3.5	*DLG4,FGFR1,FN1,ITGB3,JAK3,LAT,CLK1,CLK2,CLCF1,IGHG1,COL16A1,PLCB2*
Inorganic anion transport	−3.5	*DLG4,FGFR1,FN1,ITGB3,JAK3,LAT,CLK1,CLK2,CLCF1,IGHG1,COL16A1,PLCB2*
Phosphoric diester hydrolase activity	−3.4	*PDE2A,ENPP3,PLCB2,PDE10A,ENTPD6*
Receptor internalization, receptor-mediated endocytosis	−3.1	*ACHE,DLG4,ITGB3,ACKR3,IGHA1*
Hallmark Myogenesis, calcium ion binding	−3.1	*ACHE,COL4A2,RYR1,TNNT2,HSPB8,ENTPD6,SYT12,C1QA,DLG4,DNASE1L3,ENPP3,PLCB2,THBS2,RAB44*
***Genes with higher expression in subfertile*** **vs.** ***fertile mares***		
Monocarboxylic acid transmembrane transport activity	−5.6	*SLC10A2, SLC16A5, SLC16A9*
Protein targeting	−3.0	*RPL21,RPS17, TOMM7*

**Fig. 3:**
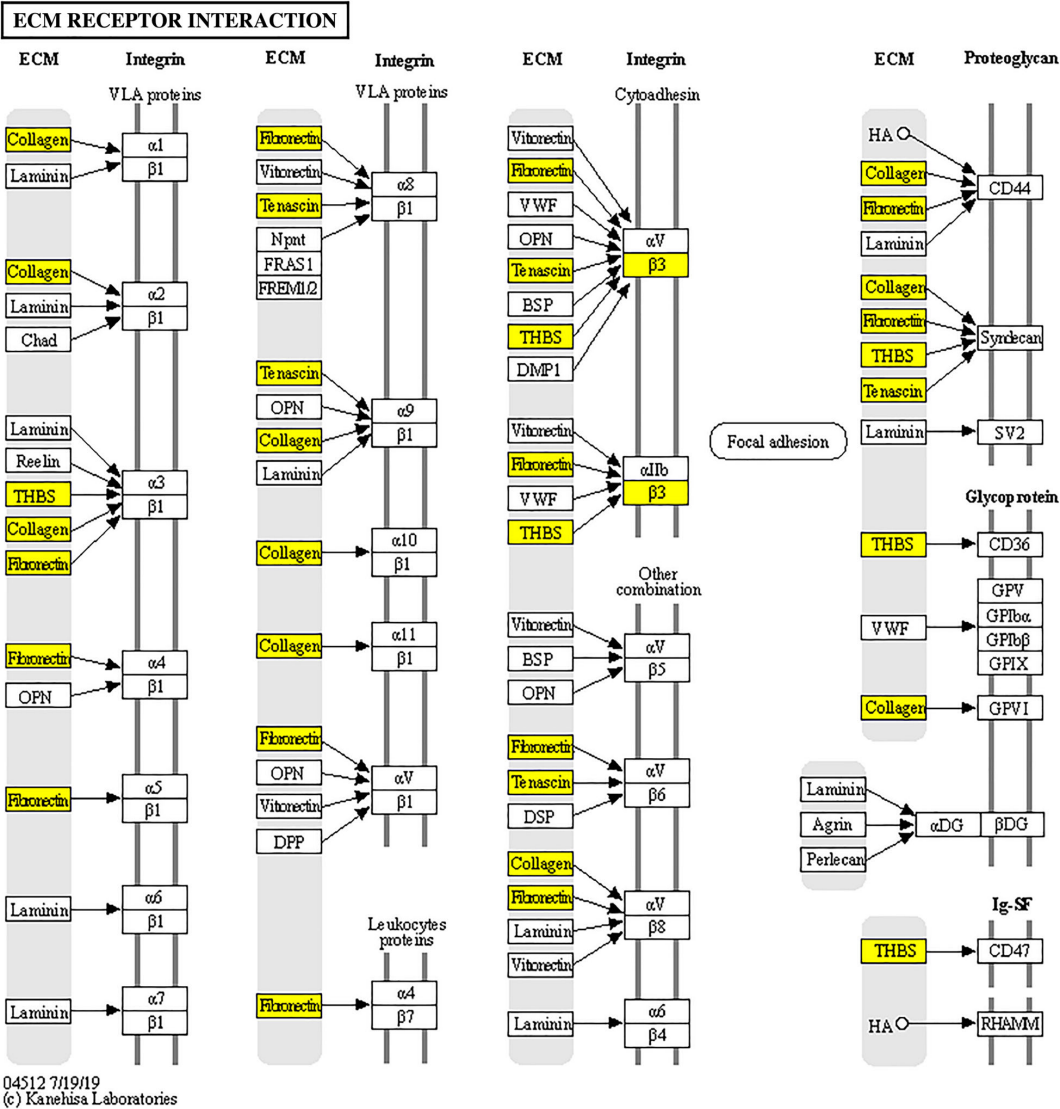
KEGG-Pathway ECM-receptor interaction: Genes belonging related to ECM-receptor interaction were significantly overrepresented for DEGs downregulated in subfertile mares (highlighted in red). Copyright permission for the KEGG pathway hsa04512 ECM-receptor interaction - *Homo sapiens* (human) has been obtained from KEGG [37]

For genes with lower expression in subfertile compared to fertile mares, functional categories such as ‘extracellular matrix (ECM)’, ‘lymphocyte mediated immunity’, ‘immune response’, ‘positive regulation of cytosolic calcium ion concentration’ and ‘peptidyl-tyrosine phosphorylation’ were found as overrepresented. The most significantly enriched KEGG pathways were ‘ECM-receptor interaction’ (Fig. 3), ‘focal adhesion’ and ‘PI3K-Akt signaling pathway’. DEGs upregulated in subfertile mares were enriched for ‘monocarboxyl acid transmembrane transporter activity’ and ‘protein targeting’.

### Validation of RNA-seq results by quantitative real-time RT-PCR

**Fig. 4:**
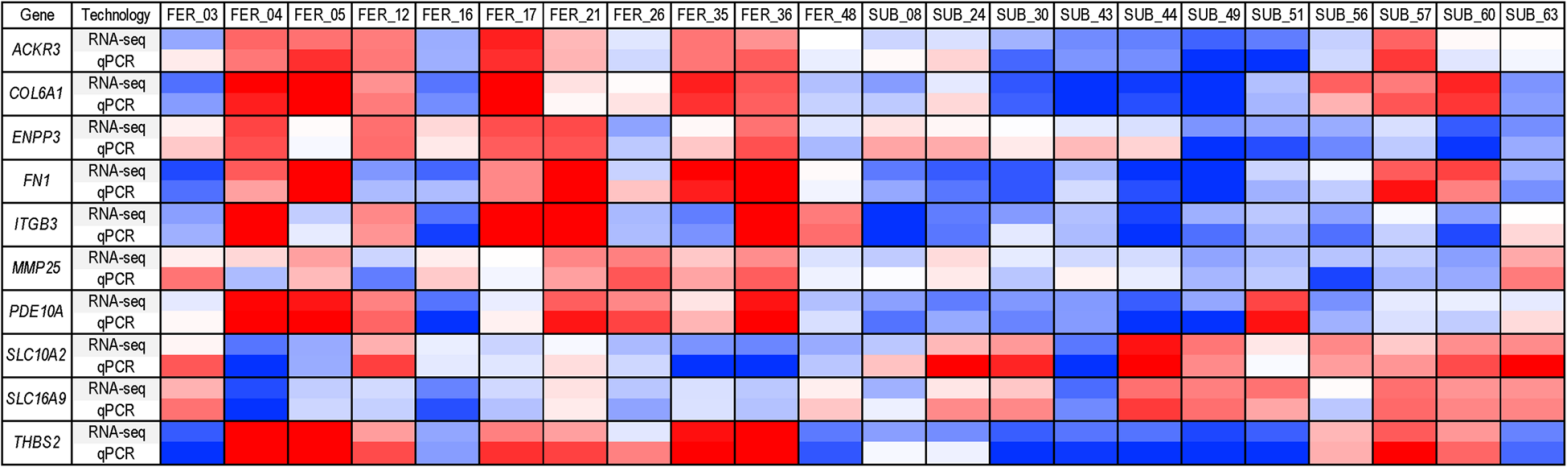
Confirmation of RNA sequencing results: Heatmap of quantitative real-time RT-PCR and RNA-seq data for ten selected genes. To illustrate correlation of RNA seq and qPCR data relative expression levels (mean-centered log2 expression values) are shown as a heatmap. Red color means higher and blue lower expression levels of the gene compared to the mean of all samples, respectively (from 2 over 0 to −2)

Expression differences found by RNA-sequencing were confirmed by qRT-PCR for 10 selected DEGs (Table 4). The qRT-PCR and RNA-seq relative expression values correlated well for the 22 analyzed samples (Fig. 4).

**Table 4:** Quantification of selected genes with quantitative real-time RT-PCR and comparison with RNA sequencing results

Gene symbol	q-RT-PCR		RNA-Sequencing	
	Log2 FC	*P*-value	Log2 FC	FDR
*SLC10A2*	1.7	0.0497	1.8	0.0368
*SLC16A9*	1.2	0.0127	1.0	0.0200
*ITGB3*	−1.9	0.0095	−1.3	0.0113
*THBS2*	−2.1	0.0269	−3.2	0.0080
*FN1*	−1.6	0.0156	−2.5	0.0065
*COL6A1*	−1.4	0.0313	−1.9	0.0460
*ACKR3*	−1.3	0.0084	−1.6	0.0242
*PDE10A*	−1.7	0.0130	−2.4	0.0080
*MMP25*	−0.7	0.0388	−1.1	0.0375
*ENPP3*	−1.2	0.0143	−2.2	0.0200

## Discussion

To our knowledge, this is the first study investigating the relationship between uterine transcriptome and fertility in mares using cytobrush samples collected during estrus. Our study showed that sufficient amounts of high-quality RNA can be isolated from uterine cytobrush samples collected from mares. All obtained RNA samples showed RINs between 8.9 and 10 and revealed a minimum of 560 ng total RNA. In contrast to biopsy samples, the cytobrush technique does not provide information about gene expression of the whole endometrium as the cytobrush tends to collect only superficial parts of the endometrium and uterine fluid. To our knowledge, there is no study that examined, which material is exactly collected by the cytobrush. However, cytological examinations of uterine cytobrush samples in mares show primarily uterine epithelial cells, white blood cells and red blood cells [17]. In our study, the cytological examination confirmed mainly uterine epithelial cells, erythrocytes and some isolated white blood cells. Comparing biopsy and cytobrush samples in cattle, stromal and endothelial cells were enriched in biopsy samples, while endometrial epithelial cells and immune cell markers were enriched in cytobrush samples [38]. A previous study in mares at the time of recognition of pregnancy showed that the strongest gene expression differences between pregnant and cyclic state are localized in the luminal epithelium [39]. Therefore, we also expected the highest differences between fertile and subfertile mares in the endometrial epithelium, which is collected with the cytobrush samples. Cytobrush samples therefore represent a less invasive sampling alternative to the biopsy sample for transcriptome analysis. However, the different sample compositions of cytobrush and biopsy samples still need to be investigated in more detail and therefore existing fertility and endometritis markers from biopsy samples cannot always be transferred to cytobrush samples.

The comparative transcriptome analysis of cytobrush samples collected during estrus revealed significant differences in the intrauterine gene expression between subfertile mares without clinical signs of reproductive diseases and normal fertile mares. Estrus was selected to allow easy sampling through the open cervix and to develop markers for the evaluation of fertility in mares before insemination based on routine cytobrush sampling. Early diagnosis of subfertile mares gives the possibility to improve the fertility of the mare with an optimized breeding management. In the present study, the mares were divided into fertile and subfertile mares according to pregnancy diagnosis after artificial inseminations during one breeding season. Mares becoming pregnant after only one artificial insemination (AI) were assumed fertile, mares that failed to conceive after at least three AIs were classified as subfertile. However, we are aware that probably not all mares classified as fertile are really fertile. Also, subfertile mares could become pregnant just by chance with the first AI and were considered as fertile in our classification. This could be also a reason why the hierarchical cluster analysis of the identified DEGs did not show a complete and clear separation of the two groups of mares into two clusters. Some mares showed intermediate expression patterns or patterns more similar to the other group. Classification after multiple AIs and pregnancy diagnosis, as in the study of Killeen et al. [40] in cattle, would have been better, but was not possible in the stud farm due to financial, logistical and ethical reasons. Moreover, we cannot exclude, if fertility was affected by the stallion, although we included only mares in our study inseminated with chilled semen from fertile stallions. Furthermore, in the subfertile mares, samples were collected after at least two unsuccessful inseminations in previous cycles, whereas in the fertile mares the samples were taken before the first insemination in the breeding season. Therefore, previous inseminations in the subfertile mares could have an influence on the intrauterine transcriptome. In addition, the small number of 11 mares per group probably limited the power of the comparative transcriptome analysis results. Further studies have to validate the DEGs found here in a larger number of samples.

In total, 114 genes were found as differentially expressed between subfertile and fertile mares. Quantitative real-time RT-PCR confirmed the results for 10 selected DEGs. The majority of the DEGs showed uniform expression in the respective fertility groups suggesting them as good candidates for RNA markers to diagnose subfertile mares without clinical signs of reproductive diseases and to predict fertility. A part of the DEGs (cluster 2 in the hierarchical cluster analysis) showed only increased expression in some of the samples of the fertile group, making these DEGs unsuitable as markers. No similarity could be found in these samples with respect to data records of these mares, which would distinguish them from the other samples. It seems likely that low mRNA levels of the genes of cluster 2 do not cause subfertility as a sole factor but may decrease fertility in conjunction with other disturbances.

According to the functional enrichment analysis, genes related to ‘extracellular matrix’, ‘ECM-receptor interaction’, ‘focal adhesion’, ‘immune response’, and ‘cytosolic calcium concentration’ may play an important role in fertility, as intrauterine gene expression levels related to these categories were lower in subfertile mares compared to fertile mares. Genes with higher mRNA concentrations in samples derived from subfertile mares were related to ‘monocarboxyl acid transmembrane transport activity’ and ‘protein targeting’. A comparison with human fertility studies is difficult as these studies were often not conducted at estrus like our study. In the following, selected DEGs potentially important for fertility are discussed.

### ECM related genes downregulated in subfertile mares

Genes with lower mRNA levels in samples collected from subfertile mares were most significantly enriched for functional categories associated with ‘extracellular matrix (ECM)’, ‘collagen trimer’, ‘extracellular matrix organization’, ‘ECM-receptor interaction’, ‘focal adhesion’, and ‘angiogenesis’. In our study the mRNA expression of 4 collagens (*COL4A1, COL4A2, COL6A1, COL16A1*), 4 ECM glycoproteins (fibronectin 1- *FN1*, thrombospondin 2 - *THBS2,* tenascin XB - *TNXB,* acetylcholinesterase (Cartwright blood group) - *ACHE*), the ECM receptor integrin subunit beta 3 (*ITGB3)* and two ECM modifiers (*ADAMTS5, MMP25)* was lower in subfertile mares compared to fertile mares. The genes *ITGB3*, *THBS2*, *TNXB*, *COL4A1, COL6A1*, *COL4A2* and *FN1* are involved in ‘ECM receptor interaction’ and ‘focal adhesion’ pathway, referring to the interaction of cells with the ECM, whereby integrins or proteoglycans establish the link between ECM and cells.

Previous studies showed that ECM-related uterine gene expression varied during the equine estrous cycle [24, 25]. Genes related to ECM, focal adhesion and angiogenesis were upregulated during estrus in equine endometrium [24, 25], which suggests an important function of these genes during estrus, that has not been clarified yet. Our study indicates that higher expression levels of genes related to ECM and focal adhesion during estrus might be important for fertility, as subfertile mares showed lower mRNA levels of these genes.

Genes related to ECM and cell adhesion were identified in fertility studies in humans at time of implantation as dysregulated genes in the endometrium of women with unexplained infertility or with recurrent miscarriage [27, 29, 41]. For instance the gene *ITGB3*, one DEG of cluster 2, is a marker of endometrial receptivity at the window of implantation in women and mice [42–44]. However, not much is known about the function of ECM related uterine genes and its relation to fertility at estrus, the time point of our sampling, as most human fertility studies are conducted during secretory phase.

Ectonucleotide pyrophosphatase/phosphodiesterase 3 (ENPP3) protein expression was only found in cyclic and not in postmenopause endometria of women indicating a relation with fertility [45]. In the study of Klein et al. [46], uterine *ENPP3* mRNA was upregulated in pregnant mares compared to cyclic mares during the time of maternal recognition of pregnancy 13.5 days after ovulation. Different DEGs are involved in ECM remodeling and collagen organization. Matrix metallopeptidase 25 (*MMP25*) and ADAM metallopeptidase with thrombospondin type 1 motif 7 (*ADAMTS7*), both involved in remodeling and breakdown of ECM, were downregulated in subfertile mares. MMP25 is able to clear the ECM components type IV-collagen, gelatin, fibronectin and fibrin in vitro [47]. The gene *TNXB* encodes the extracellular matrix glycoprotein tenascin XB. Tenascin plays a role in the organization and formation of elastin and fibrillar collagen in the ECM and is important for tissue structure and elastic fiber stability [48]. Tenascin-deficient women were reported to have a higher risk of complications during pregnancy such as preterm premature rupture of membranes [49, 50], but *Tnx* knockout mice showed no or only mild pregnancy-related abnormalities [50]. A function of *TNXB* during estrus regarding fertility was not reported yet. Thrombospondin 2 modulates collagen fibrillogenesis and inhibits angiogenesis [51]. Overall, dysregulations of ECM components involved in focal adhesion could be related to disturbances of endometrial remodeling during the estrous cycle affecting uterine interactions with the embryo and/or sperm.

### Immune-related genes downregulated in subfertile mares

Metascape analysis revealed overrepresentation of DEGs related to ‘lymphocyte mediated immunity’, ‘classical pathway of complement activation’, ‘adaptive immune response’ and ‘regulation of immune effector process’ in genes downregulated in subfertile mares in comparison to fertile mares. At the time of sampling in midestrus, 1-3 days before insemination, there was no evidence of inflammation neither clinically nor facultative pathogens in the microbial culture or positive findings in the cytological examination in the selected mares for RNA-seq. Marth et al. [25] and Gebhardt et al. [24] described in previous studies that genes associated with immune function are upregulated in the equine uterus during estrus in healthy mares. The authors suspected that the endometrium is preparing for mating during estrus and the required uterine clearance after mating with upregulation of immune-related genes and infiltration by immune cells, respectively. During estrus, the activity of the uterine immune system is increased due to intrauterine inoculation of foreign material such as bacteria, debris and sperm through the open cervix and by mating. A rapid high peak of inflammatory response after invasion of pathogens and a tight balance between pro- and anti-inflammatory factors are required to eliminate pathogens and avoid prolonged inflammation [23, 52]. After AI an inflammatory response is important to effectively remove excessive spermatozoa, seminal plasma and other contaminants from the uterus before the embryo reaches the uterus 5–6 days after fertilization [7]. For instance, the innate immune response of mares susceptible to PBIE is altered after breeding or bacterial inoculation, and characterized by abnormal imbalance in pro-inflammatory and anti-inflammatory cytokines [8, 20, 21, 23]. In our study, the fertile mares with upregulated DEGs related to immune response might therefore be better prepared for these challenges during estrus than the subfertile mares. Clinically or in rectal ultrasound, no differences between the fertile and subfertile mares could be seen in estrus or after insemination. No mares showed prolonged inflammation after insemination in rectal ultrasonic examinations [53]. Also the recently identified diagnostic markers (*DEFB1, LYZ,* and *SLPI*) for susceptibility to PBIE were not differentially expressed between subfertile and fertile mares [22], supporting the assumption that subfertility in mares was not due to susceptibility to PBIE. Whereby these diagnostic markers were developed for uterine biopsy samples and not for cytobrush samples. The mRNAs encoding cytokines, which differ in susceptible mares after insemination, were not differentially expressed at estrus in the fertile and subfertile mares.

Different genes encoding proteins involved in activation of lymphocytes were downregulated in subfertile mares. The major histocompatibility complex, class I, A (*HLA-A)* and linker for activation of T cells (*LAT)* have a role in T cell activation [54]. The genes *IGHG1* (*LOC102150085*, *LOC102149846*), *IGHA1* (*LOC106781303*), *IGHM* (*LOC100055483*), encoding heavy constants of immunoglobulins, and the DEGs cardiotrophin like cytokine factor 1 (*CLCF1)* and Janus kinase 3 (*JAK3)* are involved in B-cell activation [55, 56]. Genes encoding immunoglobulins were also lower expressed in endometrial biopsies of subfertile heifers compared to high fertile heifers on day 14 after estrus [35]. The *CLCF1* gene has been found as expressed in the equine adult ovary [56] and in equine endometrium on day 16 of pregnancy [57]. Janus kinase 3 (JAK3) mediates signaling events in innate and adaptive immunity. Mutations in the *JAK3* gene leads to severe combined immunodeficiency in humans and mice due to a disturbed T- and B-lymphocyte development and function [58, 59]. In innate immune cells, *JAK3* inhibition was reported to enhance the toll like receptor mediated production of pro-inflammatory cytokines while suppressing the anti-inflammatory cytokine IL10 [60]. Besides immune function, in vitro cultured bovine endometrial cell experiments showed that bovine endometrial cells treated with JAK3 had an increased cell viability, suggesting that JAK3/STAT pathway may also be involved in cell proliferation in endometrial cells [61].

Another downregulated gene of particular interest is the cytomegalovirus UL 16 binding protein 1 gene (*LOC100063831*), one DEG from Cluster 2. The human orthologue *ULBP3* gene encodes a ligand of the immunoreceptor NKG2D, which is mainly expressed by natural killer cells (NK) and T cells [62]. As a ligand of NKG2D of natural NK cells, ULBP stimulates cytokine and chemokine production from NK cells. Furthermore, decidual NK cells play a role in early human and mouse pregnancy by regulation trophoblast invasion and vascularization in the decidua [63–65].

Genes involved in the classical pathway of complement activation were downregulated in uterine cytobrush samples of subfertile mares. The genes *C1QA* and *C1QB* encode the A- and B-chain polypeptide of complement component C1q, which is the first component of the classical pathway of complement activation and is activated through binding of immunoglobulins [66]. Genes encoding immunoglobulins were also downregulated in subfertile mares. Besides the function in the complement pathway, C1q also possesses a physiological role in trophoblast invasion, spiral arteries remodeling and placentation in the mouse [67–69]. C1q-deficient mice showed pregnancy disorders characterized by increased fetal death and signs similar to human pre-eclampsia.

Different genes participating in regulation of innate immune cells showed lower uterine mRNA levels in subfertile mares compared to fertile mares. For instance, phosphatidylinositol-3,4,5-triphosphate dependent Rac exchange factor 1 (*Prex1)* is strongly expressed in leukocytes and is involved in neutrophil recruitment during inflammation [70]. Besides the function in extracellular matrix remodeling, MMPs also participate in regulation of inflammation and innate immunity for example by activating and degrading chemotactic molecules [71]. Previous studies showed that the expression of several MMPs (*MMP1, MMP2, MMP3, MMP7, MMP8, MMP9*) was upregulated in the endometrium of mares after inoculation of *Streptococcus zooepidemicus* or *E. coli* or after insemination [52, 72]. *MMP25*, which was differentially expressed between subfertile and fertile mares in our study, has not been described previously in the equine uterus. In mice, *Mmp25* expression has been found predominantly in peripheral blood leukocytes. It participates in the regulation of innate immune response. *Mmp25*-deficient mice are fertile but show a deficiency in their innate immune response [73]. The downregulated *ACKR3*, also known as *CXCR7* or decoy receptor D6, regulates innate and adaptive immunity with his activity as a decoy and scavenger receptor for inflammatory chemokines and thus is involved in the control of inflammation [74]. In mares, *ACKR3* is expressed significantly higher in endometrium during estrus compared to diestrus [25] and was increased after inoculation of *E. coli* [52]. *ACKR3* might play a role also in the balancing between protection of the developing embryo and tolerance of its hemiallogeneic tissues [74]. *Ackr3*-deficient mice showed an earlier and exacerbated inflammatory response in a model of skin inflammation, with high levels of inflammatory chemokines [75]. Although *Ackr3*-deficient mice were fertile, exposure to LPS or antiphospholipid autoantibodies resulted in higher levels of inflammatory CC chemokines and increased leukocyte infiltrate in placenta, causing an increased rate of fetal loss [76]. The higher level of *ACKR3* mRNA expression in fertile mares could affect fertility by preventing an excessive inflammatory response to insemination and might also be important in establishment of pregnancy.

Cysteine rich secretory protein 2 mRNA (*CRISP2*) was not strongly represented in the uterus samples; nevertheless, a significantly lower level of *CRISP2* mRNA was observed in subfertile compared to fertile mares. The gene *CRISP2* plays an important role in sperm motility, sperm capacitation and sperm-egg fusion and is positively correlated to male fertility in humans [77], mice [78], and bulls [79]. In stallions, only *CRISP3* expression correlates with stallion fertility [80]. Equine CRISP3, highly presented in seminal plasma, suppresses binding between PMNs and viable spermatozoa in the reproductive tract of the mare [81]. Cysteine rich secretory proteins expression and proteins were also found in some studies in the female reproductive tract of mice [82] and humans [83]. In vitro experiments showed that epithelial and neutrophil-derived *CRISP3* plays a role in mouse postmenstrual endometrial repair and regeneration and *CRISP3* increases adhesion and proliferation of human epithelial cells [83]. To our knowledge, it is the first time that *CRSIP2* was described in the equine endometrium. In a recently published study, Klein et al. [57] reported that *CRISP3* is expressed in conceptus tissue at day 16 of pregnancy.

Taken together, a variety of genes related to activation of lymphocytes and genes involved in regulation of immune cells were identified as differentially expressed in cytobrush samples of subfertile and fertile mares. These findings could indicate a dysregulation of the immune response in the mares of the subfertile group that might interfere with establishment of pregnancy.

### Cytosolic calcium ion concentration related genes downregulated in subfertile mares

Furthermore, DEGs were found to be involved in ‘positive regulation of cytosolic calcium ion concentration’ and ‘muscle contraction’. Calcium ion mobilization in cells occurs via Ca^2+^ channels located in the plasma membrane or via intracellular release of calcium from sarcoplasmic reticulum or acidic lysosomal stores [84]. For instance, the DEG *SLC8B1* encodes a mitochondrial Na^+^/Ca^2+^ exchanger (NCLX) [85]. The DEG two pore calcium channel 2 (*TPCN2)* encodes two pore segment channel 2 protein, a key component of the NAADP receptor, necessary for NAADP-mediated Ca^2+^ release from lysosome related organelles [84]. Intracellular Ca^2+^ release from the sarcoplasmic reticulum occurs via ryanodine receptor, encoded by the DEG *RYR1*, or inositol 1,4,5-triphospate (IP3)-receptor. Through activation of G protein linked receptor Phospholipase C ß (*PLCB2*) it hydrolyzes phosphatidylinositol 4,5-biphosphate into diacylglycerol (DAG) and IP3. Increased concentration of IP3 leads to Ca^2+^ secretion from the sarcoplasmic reticulum [86]. For example, neuromedin U, encoded by the DEG *NMU*, binds to the G-protein coupled NMU2-receptor, which activates Phospholipase C and has an uterocontractile effect [87]. In accordance with our study, another member of the neuromedin family neuromedin B was upregulated in endometrial biopsy samples of fertile cows compared to infertile ones [32]. The cytosolic calcium concentration controls processes such as metabolism, secretion, fertilization, proliferation, and smooth muscle contraction [88]. Uterine contractility in mares after insemination is important to carry sperm toward the oviduct and to eliminate excessive sperm and contaminants from uterus [89, 90].

### Genes upregulated in subfertile mares

Genes upregulated in subfertile mares without clinical signs of uterine diseases compared to fertile mares were mainly related to ‘monocarboxylic acid transmembrane transporter activity’ (*SLC10A2, SLC16A5, SLC16A9*) and protein targeting (*RPS17, RPL21, TOMM7, 60S ribosomal protein L26-like pseudogene, 40S ribosomal protein S2 pseudogene*). Several genes encoding ribosomal proteins were also more abundant in low-fertility heifers compared to high fertility heifers at day 14 post estrus [35].

The gene *SLC10A2* encodes a sodium dependent bile acid transporter highly expressed in the liver and intestine [91]. Higher abundance of *SLC10A2* were observed in bovine follicular fluid of lactating cows than in heifers suggesting that increased bile acids within follicular microenvironment may be the reason for fertility problems in lactating cows [92]. The DEGs *SLC16A5* and *SLC16A9* encode monocarboxylate transporters MCT6 and MCT9. MCT6, highly expressed in placenta and kidney, transports bumetanide, nateglinide, probenecid and PFG2α. The transport of bumetanide is pH dependent and elevated by extracellular acidic pH [93, 94]. MCT9 is involved in transport of pyruvic acid, lactic acid and carnitine [95]. The upregulated crystallin lambda 1 gene (*CRYL1)* is involved in ß oxidation of fatty acid and the upregulated ELOVL fatty acid elongase 2 (*ELOVL2)* in elongation of very long fatty acids such as arachidonic acid. Arachidonic acid in turn is a precursor of prostaglandins, which means that ELOVL2 has an influence in prostaglandin metabolism [96, 97]. In our study, higher abundance of uterine *ELOVL2* seems to have a negative effect on fertility in mares.

## Conclusions

In conclusion, cytobrush samples provide a useful source for comparative uterine transcriptome analysis in the mare, e.g., for biomarker discovery related to subfertility or other uterine diseases. Our study revealed significant differences in the uterine transcriptome at estrus between fertile and subfertile mares without clinical signs of uterine diseases. We have identified a large number of DEGs, which are potential candidates for RNA biomarkers for the prognosis of subfertility in the mare. In further studies, these results have to be validated in a higher number of mares.

## Methods

### Mares selected for the study

The study was conducted between July and October 2018 at a large private commercial stud farm in Mecklenburg-Vorpommern, Germany. In total, 57 German warmblood (Oldenburger, Hannoveraner, Westfale, Mecklenburger) mares aged between 5 and 14 years presented for routinely bacteriological examination before artificial insemination (AI) were selected for the study. Mares with foal as well as barren and maiden mares were represented. To avoid subfertility because of age related modifications of the endometrium only mares until age of 14 were included. All mares showed no clinical signs of reproductive disorders and of PBIE after previous inseminations or had no history of being susceptible to PBIE. From these 57 mares 11 fertile and 11 subfertile mares were selected for comparative transcriptome analysis. The cytobrush sample collection (for detailed description see below) was performed during the routine assessments of uterine health of the mares (such as uterine cytobrush for cytology and swab for bacteriology) owned by the Lewitz Stud, Neustadt-Glewe, Germany.

### Study design and classification as fertile or subfertile mares

**Fig. 5:**
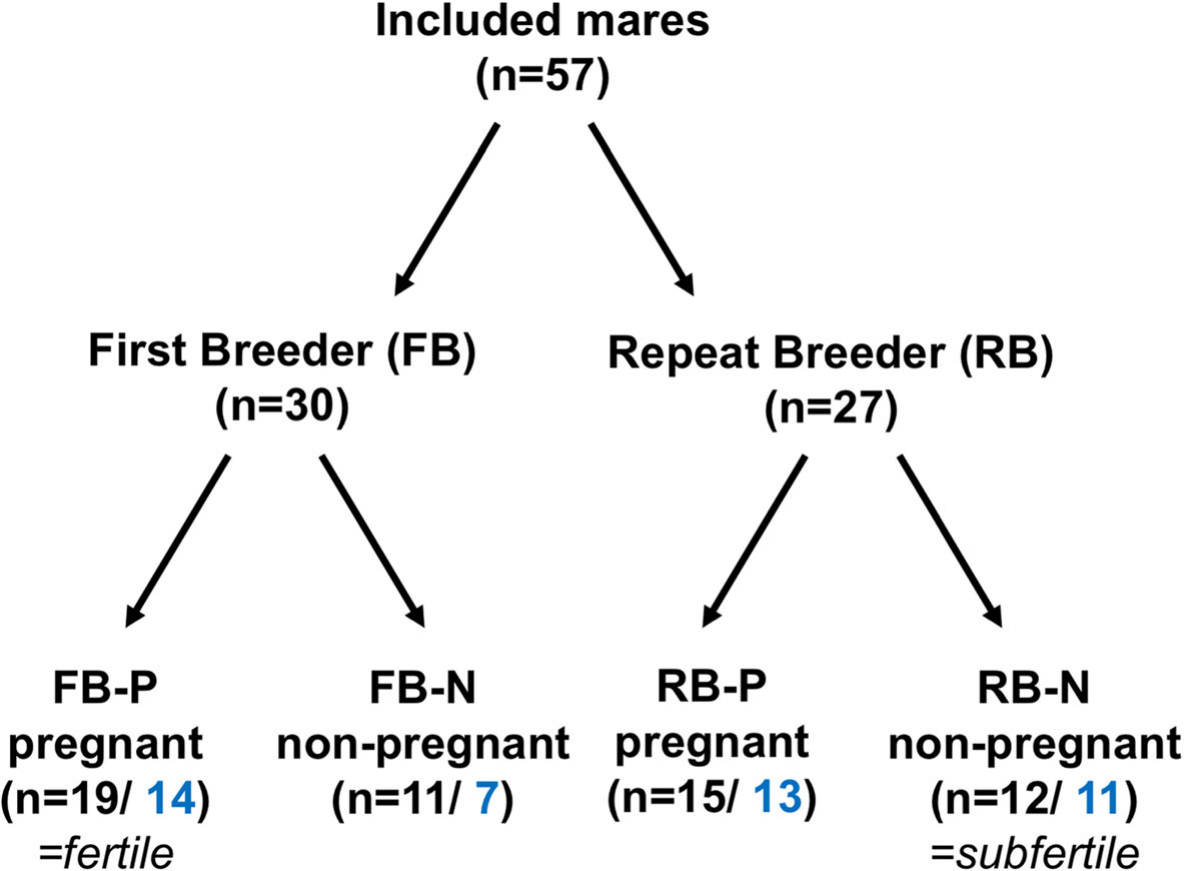
Grouping of mares according to their breeding history and fertility: First Breeder: uterine samples were taken before first AI during the season; Repeat Breeder = mares failed to conceive after at least two consecutive AIs; FB-P = mares pregnant after the first breeding during the season; FB-N = mares not pregnant after the first breeding during the season; RB-N = mares pregnant after the third breeding during the season; RB-N = mares not pregnant after at least three breedings during the season; black numbers indicate the total number of mares in each group; blue numbers indicate the number of mares after exclusion of mares with facultative pathogenic bacteria in uterine swabs. FB-P were defined fertile, RB-N subfertile. Eleven samples of FB-P and 11 samples of RB-N were used for transcriptome analysis

The 57 mares were initially subdivided according to their breeding history of the breeding season 2018 in mares having their first insemination this year (First Breeders (FB), *n* = 30) and in mares that failed to conceive after at least two consecutive AI (Repeat Breeders (RB), *n* = 27; Fig. 5). During estrus, the reproductive tract of the mares was examined daily by transrectal palpation and ultrasonography (Aloka prosound 2, Hitachi, Japan) to assess the optimal time for sampling and AI and to examine the mares for clinical signs of endometritis including intrauterine fluid, an excessive pattern of endometrial edema, vaginitis, vaginal discharge, abnormal estrous cycles and cervicitis [9, 98]. Only mares with no clinical signs for endometritis or reproductive abnormalities were included in the study. Uterine samples were collected as described later from mares in midestrus (dominant follicle > 35 mm, distinct endometrial edema). Ovulation was induced using human chorionic gonadotropin (hCG, Ovogest 1500 I.U. i.v.) when the follicle reached a diameter of > 40 mm and AI was performed 24 h later. To avoid subfertility because of low sperm quality, only chilled semen from known fertile stallions having a sperm motility > 50% were taken. Twenty-four hours after AI, the mares were examined for ovulation and intrauterine fluid by transrectal ultrasound. The first pregnancy diagnosis was carried out 16 days after single-ovulations or 14 days after double-ovulations by transrectal ultrasound. A second pregnancy diagnosis was performed 45 (+/− 2 d) days after ovulation. Mares that were positive in both pregnancy examinations were counted as pregnant. According to the results of the pregnancy examinations, mares were finally grouped into FB and RB pregnant (FB-P and RB-P) and not pregnant (FB-N and RB-N). FB-P were considered fertile (*n* = 19) and RB-N were defined subfertile (*n* = 12; Fig. 5). Mares with a positive cytological finding or facultative pathogenic bacteria in microbial culture were excluded from further analysis. Eleven mares of the subfertile group (RB-N) with no facultative pathogenic bacteria and negative cytology were used for transcriptome analysis. According to these mares, 11 fertile mares with similar age and reproductive status, negative cytology and without facultative pathogens in microbial culture were selected from the FB-P group for RNA sequencing.

### Uterine sample collection

From the 57 mares, uterine samples were taken in estrus (dominant follicle > 35 mm, distinct endometrial edema). Before sample collection, the rectum was emptied, the tail was wrapped and covered with a glove, the perineum and vulva were cleaned with soap and water and dried with a paper towel. A veterinarian (K.S.W.) wearing a sterile glove inserted manually one double guarded uterine swab (Minitube, Tiefenbach, Germany). After passing the cervix, the inner guard was advanced, and the swab was rolled gently on the endometrium for 15 s. The swab was retracted and removed together with the inner guard from the reproductive tract. The outer guard was left in the reproductive tract to avoid additional manipulation during the following sampling steps. The swab was transferred into Amies medium and sent to a laboratory specialized for bacteriology in equine reproduction (Labor Boese, Harsum, Germany) for conventional microbiological culture. A new inner guard with a cytobrush (Minitube, Tiefenbach, Germany) was inserted through the outer guard into the uterus. The brush was advanced and rolled gently on the endometrium for 15 s. The brush was retracted, removed from the reproductive tract and was inserted directly into a 1.5 ml reaction tube containing 350 μl of lysis buffer (AllPrep DNA/RNA Kit, Qiagen), rolled for 20 s and then removed. The reaction tubes were snap-frozen in liquid nitrogen until further analysis. In the same way, a second cytobrush sample was collected. The second cytobrush was rolled onto two microscope slides for cytological examination.

### Sample examination

*Cytological examination:* After air drying, the microscope slides were stained with Diff-Quick (Diff Quick, Labor+Technik Eberhard Lehmann GmbH, Germany). The slides were evaluated under a light microscope (Olympus Ch-2, Olympus, Tokyo, Japan) as described by Ferris [17]. Briefly, at 400 x magnification, 10 high power fields were analyzed, and the number of neutrophil granulocytes was counted. The presence of neutrophil granulocytes defines the following categories: no granulocytes to rare is a negative finding (healthy), 1–2 granulocytes per field indicates a mild inflammation, 3–5 moderate inflammation, > 5 severe inflammation [17].

*Bacteriological examination:* Bacteriological examination was performed at laboratory Böse (Harsum, Germany) specialized for bacteriology in equine reproduction. Bacteria were cultured on Columbia agar with 5% sheep blood, Columbia CAP Selective agar with sheep blood and Gassner agar (water blue-metachrome yellow- lactose-agar). Additionally, a mycological examination was performed on Sabouraud-Glucose agar with gentamycin and chloramphenicol and on Kimmig-agar. After incubation at 37 °C for 24 h and 48 h, the bacterial and fungal growth was evaluated. The aerobic microflora was identified by cultural-biochemical conventional methods and mass spectroscopy (MALDI-TOF). The samples were divided into mild (< 30 colonies), moderate (30–100 colonies) and severe (> 100 colonies) growth. The bacteriological results were divided into facultative pathogenic bacteria and questionable pathogenic bacteria. Growth of *ß-hemolytic Streptococcus, Pseudomonas aeruginosa, Klebsiella pneumoniae, Staphylococcus aureus, Candida albicans*, *Aspergillus spp.* and moderate to severe growth of *E. coli* were considered as facultative pathogens. Mild growth of *E. coli* and the growth of other bacteria were considered as questionable pathogens. Samples with facultative pathogens were excluded from further analysis (*n* = 12).

### Extraction of RNA and estimation of concentration and quality

The total RNA extraction was performed with the AllPrep DNA/RNA micro kit (Qiagen, Hilden, Germany) according to the manufacturer’s instructions using the protocol of Simultaneous Purification of Genomic DNA and Total RNA from Animal and Human Tissues. Because of rolling the cytobrush directly in lysis buffer after taking the sample, the initial start of the protocol was at step three of the protocol. RNA and DNA were extracted according to manufacturer’s instructions, instead of step 7 steps F1–F4 were used. RNA was eluted after two five-minute incubations with 14 μl of RNase-free water.

The total RNA concentration and purity were measured by spectrophotometry (Nano Drop One, Thermo Scientific). The quality of the isolated total RNA was assessed using the Agilent 2100 Bioanalyzer RNA 6000 Nano assay (Agilent Technologies, Waldbronn, Germany).

### RNA-sequencing and data analysis

For Illumina RNA-sequencing, 11 samples of subfertile mares (RB-N) and 11 of the fertile mares (FB-P) were selected. The isolated RNA was used for standard Illumina messenger RNA sequencing following the TrueSeq stranded mRNA protocol, which was performed by the Functional Genomic Center Zürich. The samples were barcoded and pooled and the pool was sequenced on an Illumina NovaSeq 6000 (100 bp single-end reads).

The data analysis of the resulting fastq files was performed on a local Galaxy server installation [99, 100]. In Galaxy, as first step and after every fastq file processing step, ‘Fast QC’ and ‘Multi QC’ were used to control the quality of the data and the processing steps. At first, ‘Trim Galore’ was used to trim adapter sequences, low quality ends and to discard reads shorter than 50 bp. To map the reads to the annotated equine genome equCab 3.0 downloaded from the National Center for Biotechnology Information (NCBI), the tool ‘HISAT2’ [101] was used. With the tool ‘Feature Counts’, the number of RNA sequence reads for the annotated genes were counted considering the strand specificity of the reads. ‘Column Join on data’ was used to build a count table containing all samples. This count table was filtered to remove genes with negligible read counts by using the counts per million (CPM) per sample filtering tool [102]. The mean library size and potential CPM cutoff (Counttable statistics, custom Galaxy tool) were calculated and the cutoff set to 1.09 CPM (corresponding to an average of 20 reads per library) for at least 5 out of the 22 libraries. The filtered count table was used in the statistical program R with the package edge R [103] for the identification of differentially expressed genes (DEGs) between subfertile and fertile mares. Genes with an adjusted *P*-value (false discovery rate, FDR) lower than 0.01 (FDR 10%) were considered as differentially expressed.

The web tool MAdb (Gene Symbol match, Ensembl compara database release 95, Blast) (https://madb.ethz.ch/) [104] was used to obtain the corresponding human gene symbols and gene information for the DEGs. Hierarchical clustering of DEGs was performed with the HCL tool of Multi Experiment Viewer (MeV) [105]. Metascape enrichment analysis program (http://metascape.org) [106] was used for identification of overrepresented functional categories and pathways of the DEGs. The annotation and enrichment analyses were performed separately for genes upregulated or downregulated in subfertile mares compared to fertile mares based on the corresponding human NCBI gene IDs.

The RNA-seq data presented in this study are openly available at NCBI’s Sequence Read Archive (SRA) under the BioProject accession PRJNA667444.

### Quantitative real-time RT-PCR

The same RNA samples (400 ng total RNA) as used for RNA sequencing were reverse transcribed into first-strand cDNA using the RNA to cDNA EcoDry™ (Double Primed) Premix (Takara Bio Company, USA). The cDNA samples were diluted with RNase/DNase-free water to a total volume of 40 μl.

To validate RNA sequencing results, ten DEGs with known gene symbols and with potential role in fertility were selected from the most significantly enriched Metascape categories for quantitative real-time RT-PCR. The DEGs solute carrier family 10 member 2 (*SLC10A2)*, solute carrier family 16 member 9 (*SLC16A9)*, integrin subunit beta 3 (*ITGB3)*, collagen type 6 alpha 1 chain (*COL6A1)*, thrombospondin 2 (*THBS2)*, fibronectin 1 (*FN1)*, matrix metallopeptidase 25 (*MMP25)*, ectonucleotide pyrophosphatase/phosphodiesterase 3 (*ENPP3)*, atypical chemokine receptor 3 (*ACKR3)* and phosphodiesterase 10 A (*PDE10A)* were selected. The mapping of RNA sequencing reads for these genes were checked with Integrated Genomics Viewer (IGV) [107] and matching primers were designed using Primer-Blast (NCBI) [108]. The primer sequences (ordered from Integrated DNA Technology, Leuven, Belgium) and their annealing temperatures are listed in Table 5.

**Table 5:** Primers used for quantitative real-time RT-PCR. Primers were designed using NCBI’s Primer BLAST. Ten DEGs were selected to validate RNA-sequencing results by qRT-PCR. ACTB, GAPDH and 18S rRNA were used as reference genes. The designed primers are listed with product length and annealing temperature. *F* forward primer, *R* reverse primer

Gene	Primer sequence 5′-3’	Product length (bp)	Annealing Temp C°	Accession no./reference of target transcript
*SLC10A2*	F: ATCGTTCACCTACGAGGAGC	191	66	XM_001493450.3
	R: TCACCTTGTGGAGCGATGAC			
*SLC16A9*	F: TGTTCTTTGCTGGGCTTGGA	110	68	XM_023643589.1
	R: CAGGACGCAGAAGCCACTAA			
*ITGB3*	F: GCACCCGTTACTGTCGTGAT	145	65	NM_001081802.1
	R: AGGATGGACTTTCCACTGGC			
*THBS2*	F: TGGCTGGAAAGACTACACCG	107	65	NM_001163117.2
	R: CTGAATCCGCCATGACCTGT			
*FN1*	F: GGTCGTTACTGTGGGCAACT	101	65	XM_023642280.1
	R: CCTCTCCGATGGCGTAATGG			
*COL6A1*	F: CCTCCTGGGATAAACGGCAC	184	65	XM_001488351.5
	R: ACTCGTCCATCTCTGGTCGT			
*ACKR3*	F: ATGCCTGAGTAGCCTGGAGA	113	65	XM_023642191.1
	R: GTCCTGTGGTGATGCAAACG			
*LOC100073089 (ENPP3)*	F: TAGAATACGTGGTCAACACCAG	190	68	XM_023651094.1
	R: TCAACCCAGTTGGCTTCCTG			
*PDE10A*	F: GCGTGAATTGTAGCAGCCAG	76	65	XM_023633145.1
	R: ACTGATTGCAGAAAGACACTTCC			
*MMP25*	F: ATGTCACCGTCAGCAACACAG	189	70	XM_023633145.1
	R: GTCCAGGCTTGAGAGTGGCT			
*ACTB*	F: TCCCAGCACGATGAAGATCAA	189	68	XM_023655002.1
	R: GGTGGATCGCACTAACAGT			
*GAPDH*	F: ATTGCCCTCAACGACCACTT	140	70	NM_001163856.1
	R: TCTTGCTGGGTGATTGGTGG			
*18S rRNA*	F: GCGTGTGCCTACCCTACGCC	165	68	AJ311673.1/ [24]
	R: ATCGTTCACCTACGAGGAGC			

The mRNA expression of the selected genes was measured by real-time PCR on a Light Cycler 96 (Roche, Mannheim, Germany) with the KAPA HiFi HotStart PCR Kit (Roche, Kapa Biosystems Pty, South Africa) adding EvaGreen® Dye, 20x in water (Biotium). The qPCR was performed in a reaction volume of 10 μl, consisting of 2 μl 5x Kapa HiFi Buffer, 0.3 μl Kapa dNTP mix (0.3 mM each), 0.2 μl Kapa HiFi HotStart DNA polymerase, 0.3 μl of each primer (10 μM), 0.5 μl Eva Green Dye, 5.4 μl water and 1 μl cDNA template. Cycle parameters of the PCR were 95 °C for 3 min, followed by 45 cycles of 98 °C for 20 s, specific annealing temperature for 15 s and 72 °C for 15 s, and then a melting step (95 °C for 10 s, 65 °C for 60 s and 97 °C for 1 s). Melting curves of the amplified PCR products were obtained for confirmation of specific amplification. A no-template control (RNA sample) was included for each primer pair. The C_q_ (quantification cycle) values determined for the target genes were normalized against the geometric mean of the reference genes beta actin (*ACTB*), glyceraldehyde-3-phosphate dehydrogenase (*GAPDH*) and 18S rRNA [109]. Relative expression differences between subfertile and fertile mares were calculated, and a t-test was performed in Microsoft Excel. *P*-values < 0.05 were considered significant.

## Supplementary Information

**Additional file 1.** Differentially expressed genes (DEGs) in cytobrush samples collected from subfertile vs. fertile mares.

**Additional file 2.** Metascape enrichment analysis results of transcripts downregulated or upregulated in cytobrush samples collected from subfertile vs. fertile mares.

## Data Availability

The datasets generated during the current study are in this published article and the RNA-seq data are openly available at NCBI’s Sequence Read Archive (SRA) under the BioProject accession PRJNA667444 https://www.ncbi.nlm.nih.gov/sra/?term=PRJNA667444.

## Abbreviations

AI: Artificial insemination
CPM: Counts per million
DEG: Differentially expressed gene
DNA: Deoxyribonucleic acid
cDNA: Complementary DNA
ECM: Extracellular matrix
FB: First Breeders
FB-P: First Breeder-pregnant
FB-N: First Breeder-not pregnant
FDR: False discovery rate
i.v.: Intravenous
KEGG: Kyoto Encyclopedia of Genes and Genomes
Log2 FC: Log2-fold-change
mRNA: Messenger ribonucleic acid
NCBI: National Center for Biotechnology Information
NAADP: Nicotinic acid adenine dinucleotide phosphate
NKG2D: Natural Killer Group 2D
PBIE: Persistent breeding-induced endometritis
RB: Repeat Breeders
RB-P: Repeat Breeder- pregnant
RB-N: Repeat Breeder- not pregnant
RIN: RNA integrity number
RNA: Ribonucleic acid
RNA-seq: RNA sequencing
RT-PCR: Reverse transcription- Polymerase Chain Reaction
qRT-PCR: Quantitative RT-PCR
